# Evaluation of the Indian Migration Study Physical Activity Questionnaire (IMS-PAQ): a cross-sectional study

**DOI:** 10.1186/1479-5868-9-13

**Published:** 2012-02-09

**Authors:** Ruth Sullivan, Sanjay Kinra, Ulf Ekelund, Bharathi AV, Mario Vaz, Anura Kurpad, Tim Collier, K Srinath Reddy, Dorairaj Prabhakaran, Shah Ebrahim, Hannah Kuper

**Affiliations:** 1 Department of Epidemiology and Population Health, London School of Hygiene & Tropical Medicine, London, UK; 2 MRC Epidemiology Unit, Institute of Metabolic Science, Cambridge, UK; 3 Mount Carmel College, Indira Gandhi National Open University, Bangalore, India; 4 Division of Nutrition, St John's Research Institute, Bangalore, India; 5 Division of Health and Humanities, St John's Research Institute, Bangalore, India; 6 Public Health Foundation of India, New Delhi, India; 7 Centre for Chronic Disease Control, New Delhi, India; 8 South Asia Network for Chronic Disease, Public Health Foundation of India, New Delhi, India

**Keywords:** Health behaviour, Activity Domains, Low-Middle Income Countries, Reproducibility, Adults, Methodology

## Abstract

**Background:**

Socio-cultural differences for country-specific activities are rarely addressed in physical activity questionnaires. We examined the reliability and validity of the Indian Migration Study Physical Activity Questionnaire (IMS-PAQ) in urban and rural groups in India.

**Methods:**

A sub-sample of IMS participants (n = 479) was used to examine short term (≤1 month [n = 158]) and long term (> 1 month [n = 321]) IMS-PAQ reliability for levels of total, sedentary, light and moderate/vigorous activity (MVPA) intensity using intraclass correlation (ICC) and kappa coefficients (k). Criterion validity (n = 157) was examined by comparing the IMS-PAQ to a uniaxial accelerometer (ACC) worn ≥4 days, via Spearman's rank correlations (ρ) and k, using Bland-Altman plots to check for systematic bias. Construct validity (n = 7,000) was established using linear regression, comparing IMS-PAQ against theoretical constructs associated with physical activity (PA): BMI [kg/m^2^], percent body fat and pulse rate.

**Results:**

IMS-PAQ reliability ranged from ICC 0.42-0.88 and k = 0.37-0.61 (≤1 month) and ICC 0.26 to 0.62; kappa 0.17 to 0.45 (> 1 month). Criterion validity was ρ = 0.18-0.48; k = 0.08-0.34. Light activity was underestimated and MVPA consistently and substantially overestimated for the IMS-PAQ vs. the accelerometer. Criterion validity was moderate for total activity and MVPA. Reliability and validity were comparable for urban and rural participants but lower in women than men. Increasing time spent in total activity or MVPA, and decreasing time in sedentary activity were associated with decreasing BMI, percent body fat and pulse rate, thereby demonstrating construct validity.

**Conclusion:**

IMS-PAQ reliability and validity is similar to comparable self-reported instruments. It is an appropriate tool for ranking PA of individuals in India. Some refinements may be required for sedentary populations and women in India.

## Background

Evidence derived mainly from observational studies undertaken predominantly in High Income Countries (HICs), has established that there are clear health benefits from a physically active lifestyle irrespective of age, gender, ethnicity or geographic location [1,2]. Increased levels of physical activity (PA) are associated with lower blood pressure, reduced body fat, increased lean body mass, improved weight control, reduced central adiposity, enhanced musculoskeletal health and improved glucose metabolism [3,4].

The role of low physical activity as an important independent risk factor for many Non-communicable Diseases (NCD) has been well documented in the Western population,[3,5,6] yet there still remains a lack of evidence from population specific studies in Low and Middle Income Countries (LMICs) such as India. Whilst international questionnaires such as the International Physical Activity Questionnaire (IPAQ) and Global Physical Activity Questionnaire (GPAQ) have been proven to be valid and reliable,[7-9] their structure and design may limit more detailed information being gathered on regional-specific activities within rural and urban areas and across multiple domains (e.g. country-specific sports or household chores such as watering walls and making cow-dung cakes). Additionally, international questionnaires are rarely able to address the socio-cultural differences in PA that exist in large and diverse countries such as India and they often require participants to rate their own level of activity intensity based on perceived levels of exertion which in turn has been proven to be problematic within an Indian setting [10-12]. The Indian Migration Study (IMS) Physical Activity Questionnaire (PAQ) was therefore specifically designed and adapted from a pre-existing Indian activity questionnaire^14 ^to allow for the collection of PA data within both rural and urban areas across India [13].

The IMS-PAQ had previously been validated on a small convenience sample of residents (n = 13 & n = 94) based in Bangalore, southern India [14]. Our present study was therefore undertaken to provide a definitive assessment of the reliability and validity of the IMS-PAQ in India within the context of the IMS for urban and rural participants. If shown to be reliable and valid, the IMS could provide an important contribution to PA monitoring and assessment as suggested by the WHO Global Strategy for Diet, Physical Activity and Health [4] specifically within India and more generally across south Asia.

## Methods

The design and sampling methodology of the IMS has been described previously [15]. In summary, the IMS was conducted from March 2005 to December 2007 and was nested within a cardiovascular disease surveillance system focusing on four industry-based populations within India (Lucknow, Nagpur, Hyderabad and Bangalore). Factory workers and their co-resident spouses were asked to provide information on rural-to-urban migration and family status (existence, age, gender and location of living siblings). Those responding positively, along with a 25% random sample of urban non-migrants were asked to participate in the study. Each participant was asked to identify one non-migrant full sibling, preferably of the same gender and closest in age to them, who was invited to participate in the study. If no full-sibling was available a cousin (3.5%) or close friend/more distant relative (0.3%) from the same village (or city for urban non-migrant sibling-pairs) was invited. The study obtained ethics approval from the All India Institute of Medical Sciences Ethics Committee and the London School of Hygiene and Tropical Medicine. All participants provided written informed consent or a witnessed thumb-print if illiterate.

### Participants

**Figure 1:**
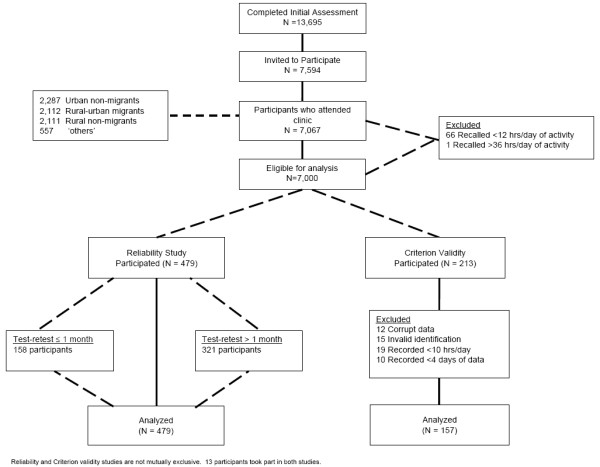
**Consort diagram for the physical activity reliability and validity sampling framework of the Indian Migration Study Questionnaire (IMS-PAQ)**.

The response rates of the IMS have been reported previously [15]. A schematic of the sampling framework can be seen in Figure 1. From a total of 13,695 participants who completed a study eligibility assessment, 7,067 completed the clinical questionnaire. Sixty seven people (0.9% of the total sample) were excluded from subsequent analysis as they either recalled less than 12 hours of activity a day (66 participants), or over-reported by more than 12 hours a day (1 participant). The characteristics of the 7,000 participants eligible for this study are presented in Table 1.

**Table 1:** Characteristics of the Indian Migration Study participants by sub-sample type

	**Total Sample** **(Construct Validity)** **(n = 7,000)**		**Reliability Sample** **(n = 479)**		**Criterion Validation Sample** **(n = 157)**	
			
Residence N, (%)						
Urban	4,436	(63%)	302	(63%)	115	(73%)
Rural	2,564	(37%)	177	(37%)	42	(27%)
						
Sex N, (%)						
Men	4,102	(59%)	274	(57%)	120	(76%)
Women	2,898	(41%)	205	(43%)	37	(24%)
						
Age (yr), Mean (SD)	40.7	(10.3)	42.3	(10.0)	41.5	(9.5)
						
Anthropometry, Mean (SD)						
Weight (kg)^1^	61.3	(12.5)	62.0	(12.1)	64.2	(13.1)
BMI (kg/m^-2^)^1^	23.8	(4.5)	24.1	(4.4)	24.1	(4.3)
Percent Body Fat^2^	26.9	(8.2)	27.8	(7.9)	25.8	(7.2)
						
Blood Pressure^3^, Mean (SD)						
Systolic (mm Hg)^3^	122.2	(17.3)	125.2	(18.4)	124.5	(15.6)
Diastolic (mm Hg)^3^	77.9	(11.0)	79.6	(11.2)	79.2	(10.8)
						
Prevalence Diabetes % (SD)^4^	9.9	(29.8)	10.0	(30.1)	11.5	(32.0)

Data presented are frequency and percentage (%)

^1^Based on 6,996 participant in total sample; 4 missing weight data.

^2^Based on 6,869 participants in total sample; 131 missing skin-fold data.

^3^Based on 6,994 participants in total sample; 6 missing pulse rate data.

^4^Based on 6,977 participants in total sample; 23 missing information on diabetes. Diabetes mellitus, self-reported or fasting blood glucose level ≥7 mm (ol/l)

### The Indian Migration Study Physical Activity Questionnaire

The IMS-PAQ was created by modifying an existing questionnaire and was specifically designed to capture activity patterns and levels across multiple domains relevant to both rural and urban locations in India and across gender as numerous activities are gender specific in India [13,14,16]. The IMS-PAQ was administered by trained interviewers at each study site to gather information on participant's habitual PA. The PAQ took an average of 10 minutes to complete and consisted of predominantly open questions which ensured that participants were able to report on all activities undertaken (maximum 42 different activities) over the last one-month within specified domains (occupational, household, hobbies, exercise, sedentary behaviours [such as television viewing, chatting to friends, listening to the radio], travel, discretionary and sleep). For each activity, additional information was collected on its frequency and duration. Metabolic equivalent unit values (METs) were assigned to each activity using the Compendium of Physical Activity and WHO/FAO/UN guidelines, [17,18] supplemented with country-specific values [19]. One MET is equivalent to resting metabolic rate of approximately 3.5 mL of O_2_/kg/min, or 1 kcal/kg/hour, corresponding to the resting metabolic rate of sitting quietly.

Habitual daily activity was estimated for participants reporting between 12 and 36 hours of activity a day, using information recalled on activity duration and frequency over last one month e.g. time spent watching television (minutes/day). Individual daily activity durations were then summed to generate total daily duration for all reported activities. If this value equated to less than 24 hours, a residual time variable was generated and a standard MET value of 1.4, was applied as in previous studies [14,16]. Individuals over-reporting time spent in daily activities (i.e. > 24 hours/day) had the duration of each individual activity reduced proportional to the amount over-reported. Total activity was calculated as total MET (hr/day) by summing daily MET values of all activities. For occupational activities considered 'more strenuous than walking,' the Integrated Energy Index (IEI) was applied to correct total METs [20]. This adjusts for unreported rests which occur when participants recall strenuous occupational activities such as digging, which are too physically demanding to occur for prolonged periods without short breaks.

Duration of PA within different activity intensity categories from self-report were calculated using previously published intensity thresholds; sedentary < 1.5 METS; light 1.5 < 3 METS; moderate 3-6 METS; vigorous > 6 METS [1]. As only 3% of the sample reported participation in vigorous activity, moderate and vigorous activity was subsequently regrouped as moderate/vigorous physical activity (MVPA). Activity intensity categories for the IMS-PAQ could include activities from any activity domain, dependent upon the MET value of the specific activity.

### Anthropometric and Blood Pressure Measurements

Trained personnel measured height and weight during the clinic visit. Height was measured twice to the nearest 0.1 cm using a portable stadiometer with a base plate (Leicester height measure, Chasmors Ltd, London). Weight was measured twice, to the nearest 0.1 kg using a digital scale (Model PS16, Beurer, Germany), with participants removing their shoes and wearing light clothing. The mean of measures was used for analyses. Blood pressure and pulse rate were measured twice in the sitting position using the right arm with the appropriately sized cuff after a rest period of 5 minutes, using an Omron M5-I automatic machine. The mean value was used in subsequent analyses. Skinfold thickness was measured 3 times at the triceps and subscapular using Holtain callipers (Crosswell, UK), with the mean values used to estimate percent body fat using a standard formula [21].

### Testing for Reliability and Validity

#### Reliability

The IMS-PAQ was repeated in a sample of 479 individuals selected purposively (target of 1 in 20 participants, equal numbers of men and women, rural and urban) each week and covered all the four study sites. The time span between baseline and retest ranged from two weeks to over two years. Analysis was run separately for those tested within one month of initial questionnaire (n = 158) and those tested at more than one month (n = 321) as variation in reported activity from the latter group was more likely to reflect real change in activity pattern and indicate the stability of the questionnaire, rather than its reliability.

#### Criterion Validity

Participants within the IMS were asked to wear an Actigraph 7164^® ^uniaxial accelerometer for at least 4 full days during waking hours. Accelerometers were initialised to monitor and record data in 60 s epochs as 'activity counts' based upon the magnitude of vertical acceleration and were worn on the hip (right side). Duration (minutes per day) spent in different activity intensities; light (1.5 < 3 METS, 100≤1951 counts), moderate (3-6 METS, 1952 -5724 counts), vigorous (> 6 METS, ≥5725 counts) were determined according to published data [22,23]. Moderate and vigorous activity were subsequently regrouped as moderate/vigorous activity (MVPA). Time spent in sedentary activity was not analysed as part of the test for criterion validity, as it was too difficult to separate from sleep time for many participants who wore their accelerometer at night time. Only participants with at least 10 hours of activity per day (where the accelerometer measures > 0 cpm) recorded for a minimum of 4 days were included in the analysis. Two hundred and thirteen participants agreed to participate; 56 participants (26.3%) were excluded from further analysis for criterion validity; 12 (5.6%) had corrupt data, 15 (7.0%) had invalid identification information, 19 (8.9%) recorded less than 10 hours of activity a day and 10 (4.7%) recorded less than 4 days of accelerometer data. One hundred and fifty seven participants, 24% female, 27% rural, mean age 41.5 (SD 9.5 yrs; range 20-62 yrs) were included in the criterion validation part of the study (Table 1).

#### Construct Validity

All 7,000 participants who completed the IMS clinical questionnaire were included when examining the construct validity of the IMS questionnaire (Table 1).

### STATISTICAL ANALYSIS

Data were analysed using STATA 11 for Windows software. Results are presented as means and 95% Confidence Intervals (95%, CI) for normally distributed data or as geometric means and 95% CI for non-normally distributed physical activity variables. Correlation coefficient values: < 0.20 = weak correlation, 0.21-0.40 = fair correlation, 0.41-0.60 = moderate correlation, 0.61-0.80 = strong correlation and 0.81-1.0 = very strong correlation were used within this study.

Reliability was investigated through intraclass correlation based on a one-way analysis of variance of PA variables reported at baseline and retest,[24-26] presented by urban/rural status and gender. Reliability was additionally assessed by testing the ability of the questionnaire to correctly group individuals based on categories of activity intensity through kappa statistic [27]. This was achieved by creating four separate groups within each level of activity intensity (sedentary, light, MVPA), based upon quartiles of duration spent in that intensity at baseline. The same activity intensity cut points were then applied to the retest data. The group to which individuals were assigned at baseline and retest was then compared using kappa statistic. Sensitivity analysis was run comparing individuals retested within one month and those retested at more than one month from baseline. The reliability of the PAQ to measure time spent watching television as a separate sedentary activity was additionally analysed as it represented the primary sedentary behaviour of interest.

To assess criterion validity, total duration (minutes/day) of time spent in light and moderate/vigorous activity as estimated from the self-reported questionnaire (using previously described cut-points) were compared against those recorded by the Actigraph 7164^® ^accelerometer using established cut-points [23]. Accelerometer data were initially downloaded and processed using customised 'MAHUFFE' software (Cambridge, UK http://www.mrc-epid.cam.ac.uk/Research/Programmes/Programme_5/InDepth/Programme%205_Downloads.html) Additionally, total counts from the Actigraph 7164^® ^accelerometer were compared against total activity (MET hr/day [excluding sleep]) from the questionnaire. Kappa statistic was applied to test the ability of the PAQ to correctly rank individuals based on categories of activity intensity compared to the accelerometer. Four separate groups based upon quartiles of each activity variable (total METS, time spent in light and MVPA), were generated for the PAQ and accelerometer separately and kappa statistic applied.

Modified Bland Altman plots were used to graphically check for any systematic error in data reporting in the questionnaire vs. the accelerometer [28].

Construct validity was estimated by fitting linear regression models to the data to identify the relationship between tertiles of activity intensity and BMI, percent body fat and pulse rate. Tertiles of PA intensity were produced for each intensity category (sedentary, light, MVPA) with the lowest group (least amount of time spent in sedentary/light or MVPA) representing the reference group. For sedentary activity, group 3 would therefore represent the most sedentary participants and for light and MVPA it would represent individuals reporting most time in light intensity activity or MVPA. In order to test the independent effect of total activity and activity intensity (sedentary, light, MVPA), with BMI, percent body fat and pulse rate, over and above that it is related to shorter amounts of time in other activity intensities, regression was run adjusting for age, sex, migrant status and time spent in other PA intensities (total, sedentary, light, MVPA). Robust standard errors were applied to account for the clustered nature of the data (sibling-pairs). Wald tests were performed on model parameters.

#### Quality assurance

All protocols and equipment were pilot tested prior to the study commencing. Fieldworkers at the four study sites underwent training and standardisation at the outset and subsequently every six months. Anthropometric instruments were calibrated at the start of each clinic session. Application of MET values to individual activities were applied centrally to minimize data entry bias across centres.

## Results

### Study population

We have presented the characteristics of our study population for the three sub-studies (reliability, criterion and construct validity) in Table 1. Overall, each group was fairly consistent for anthropometrics and blood pressure and the small variations reported are likely to be due to the higher proportion of men in both the validity sub-sets. For the reliability sub-study we examined 479 participants, 37% of whom were rural, 43% women, with a mean age of 42.3 years (SD, 10.0 yrs; range 20-79). For the criterion validity population we examined 157 participants, 27% of whom were rural, 24% women, with a mean age 41.5 (SD 9.5 yrs; range 20-62 yrs). For the construct validity population we examined 7,000 participants, 37% of whom were rural, 41% women, with a mean age of 40.7 years (SD 10.3 years).

### Physical Activity Characteristics

PA characteristics for participants tested within one month of initial test at baseline and retest are displayed in Table 2. Individuals reported most time in sedentary activity and least time in MVPA at both baseline test and retest. These findings held true when stratified by urban/rural status and gender. Greater total activity, time spent in MVPA and sedentary activity was reported at retest compared to baseline for all groups. There was no difference in self-reported PA at baseline between urban and rural participants whereas rural participants reported more activity at retest (urban: 39.1 MET hr/day, [95% CI 38.3, 39.9]; rural: 41.8, [95% CI 40.0, 43.6]). Men reported more activity than women and at both baseline and retest (total activity MET hr/day at baseline, men 40.2 [95% CI 39.2, 41.2]; vs. women 37.4 [95% CI 36.5, 38.3]). Reported television viewing was consistent across all groups except in women where it was higher than men at both baseline and retest. These patterns of PA broadly held true for participants tested at > 1 month after initial baseline (data not shown).

**Table 2:** Physical Activity Characteristics, (mean & 95% CI)^a ^of individuals retested within one month of first test (n = 158)

	Total Activity (MET hr/day)		Sedentary Activity (min/day)		Light Activity (min/day)		MVPA (min/day)		Television Viewing (min/day)	
	**Test**	**Retest**	**Test**	**Retest**	**Test**	**Retest**	**Test**	**Retest**	**Test**	**Retest**

Total sample (n = 158)	39.3 (38.5-40.0)	39.8 (39.0-40.5)	489 (465-514)	515 (490-539)	359 (240-379)	319 (301-337)	167 (144-189)	131 (114-152)	70 (60-82)	68 (59-79)
										
Urban (n = 117)	38.7 (37.9-39.4)	39.1 (38.3-39.9)	507 (480-534)	542 (515-570)	374 (352-397)	321 (299-343)	103 (87-121)	113 (95-133)	69 (58-83)	69 (58-81)
Rural (n = 41)	40.9 (38.9-42.9)	41.8 (40.0-43.6)	439 (386-492)	436 (388-485)	317 (279-355)	313 (279-348)	201 (156-259)	202 (158-259)	73 (53-100)	67 (47-95)
										
Men (n = 103)	40.2 (39.2-41.2)	40.7 (39.7-41.6)	483 (453-515)	512 (480-543)	327 (304-351)	296 (273-318)	170 (148-196)	180 (157-206)	59 (50-69)	61 (51-72)
Women (n = 55)	37.4 (36.5-38.3)	38.1 (37.1-39.2)	500 (459-540)	520 (480-561)	420 (391-449)	363 (334-392)	61 (48-77)	72 (55-94)	97 (73-130)	84 (64-110)

^a ^Data in the table is presented as mean and 95% confidence intervals (CI), except for MVPA and television viewing which are geometric means and 95% CI.

Sedentary activity < 1.5 METS, Light activity 1.5-3 METS, MVPA > 3 METS. MVPA (Moderate & Vigorous Physical Activity)

### Reliability

Table 3 shows the test-retest reliability of the IMS-PAQ for those retested within one month of first test (n = 158). Total Activity (MET hr/day) reliability was good (ICC 0.84). Reliability was highest for MVPA, for the sample as a whole and when stratified by urban/rural status and sex. An exception was for women where TV viewing had the greatest reliability. For the sample as a whole, ICC values ranged from 0.42 for light intensity activity to 0.88 for MVPA (P < 0.001). Values were comparable and good for urban (ICC 0.55 to 0.85) and rural (ICC 0.55 to 0.91) participants with the exception of light activity, which was weak for both groups. Reliability was higher for men (ICC 0.59 to 0.89) than women (ICC 0.10 to 0.62); was generally fair or moderate (ICC ≥0.60) for MVPA, total activity and television viewing. Reliability was fair or better for sedentary activity (ICC 0.55 to 0.79) except for women (ICC 0.25). Overall, reliability was lower in women for all activity categories except television viewing (ICC women 0.62; men 0.59). The same pattern of reliability was seen when using kappa statistic with fair to substantial agreement for the sample as a whole (0.37 to 0.61), for both urban and rural participants and by gender. The reliability of the IMS-PAQ followed a similar pattern when participants seen at more than one month from initial test were analysed with lowest values for light activity and highest for MVPA and total activity. Values for both ICC and Kappa were lower although values still remained credible for the sample as a whole (ICC 0.26 to 0.62; kappa 0.17 to 0.45). Rural participants displayed higher reliability (ICC 0.25 to 0.64) than urban participants (ICC 0.26 to 0.47) with values being slightly lower in women.

**Table 3:** Test-Retest Reliability of individuals retested within one month of first test (n = 158)

	**Total** **(n = 158)**		**Urban** **(n = 117)**		**Rural** **(n = 41)**		**Men** **(n = 103)**		**Women** **(n = 55)**	
					
	**ICC**	**Kappa**	**ICC**	**Kappa**	**ICC**	**Kappa**	**ICC**	**Kappa**	**ICC**	**Kappa**
					
Total Activity (MET hr/day)	0.84**	0.58**	0.77**	0.51**	0.91**	0.74**	0.89**	0.68**	0.60**	0.36
										
Activity Intensity^1^										
Sedentary (min/day)	0.62**	0.49**	0.55**	0.44**	0.55**	0.55**	0.79**	0.59**	0.25*	0.28
Light (min/day)	0.42**	0.37**	0.43**	0.38**	0.33*	0.31*	0.59**	0.52	0.10	0.12
MVPA (min/day)	0.88**	0.61**	0.85**	0.57**	0.89**	0.65**	0.89**	0.59**	0.55**	0.39
										
TV Viewing (min/day)	0.64**	0.52**	0.66**	0.52**	0.60**	0.52**	0.59**	0.47**	0.62**	0.51

Data presented are intraclass correlation coefficient (ICC) and Kappa statistic.

Kappa coefficients are based on quartiles of physical activity intensity categories and are weighted

^1 ^Sedentary activity < 1.5 METS, Light activity 1.5-3 METS, MVPA > 3 METS. MVPA (Moderate & Vigorous Physical Activity)

P-value is a test of non-independence between the two data measures. *P < 0.05; **P < 0.001

### Criterion Validity

Table 4 displays PA data from the self-reported questionnaire and the objectively measured accelerometer (n = 157). The IMS-PAQ underestimated time spent in light intensity activity compared with accelerometry measured time for the sample as a whole and for all sub-populations except women. MVPA was consistently and substantially overestimated in the IMS-PAQ compared to accelerometry in all groups (Table 4). Bland-Altman plots suggested there is evidence of systematic bias in data reporting within this population for both light activity (minimal error) and MVPA (where a greater level of participation was associated with an increased over-estimation of self-reported activity).

**Table 4:** Criterion validity (spearman rank and kappa coefficient)^a ^and physical activity characteristics (mean and 95% CI)^b ^of IMS participants by gender and urban/rural status.

	**Total Activity**^**1**^				**Light Activity**^**2**^				**Moderate/Vigorous Physical Activity**^**2**^			
			
	**Questionnaire** **(MET hr/day)**	**Accelerometer** **(total counts per day)**	**Ρ**	**Kappa**	**Questionnaire** **(min/day)**	**Accelerometer** **(min/day)**	**ρ**	**Kappa**	**Questionnaire** **(min/day)**	**Accelerometer** **(min/day)**	**ρ**	**Kappa**
			
Total sample (n = 157)	32.3 (31.6 - 33.1)	340,506 (318,676 - 363,336)	0.41**	0.34**	362 (341 - 381)	392 (379 - 406)	0.17*	0.08	128 (113 - 146)	26 (22 - 30)	0.48**	0.32**
												
Urban (n = 115)	32.0 (31.2 - 32.9)	334,853 (308,396 - 361,310)		0.33*	361 (338 - 385)	387 (370 - 403)	0.14	0.07	119 (102 - 138)	25 (21 - 30)	0.48**	0.34**
Rural (n = 42)	33.2 (31.5 - 34.9)	355,983 (317,072 - 394,895)	0.44**	0.35**	361 (321 - 401)	408 (383 - 433)	0.31*	0.13	158 (124 - 201)	27 (20 - 36)	0.48**	0.25**
												
Men (n = 120)	33.0 (32.1 - 33.9)	357,664 (332,497 - 382,832)	0.40**	0.33**	0.41**	390 (375 - 405)	0.08	0.03	167 (149 - 186)	33 (28 - 38)	0.37**	0.24**
Women (n = 37)	30.2 (29.0 - 31.1)	284,857 (244,225 - 325,490)	0.28	0.30**	438 (392 - 484)	401 (368 - 433)	0.45*	0.20*	52 (41 - 68)	12 (8 - 17)	-0.01	0.06

^a ^Criterion validity data in the table is presented as: ρ = spearman rank correlation coefficient and kappa coefficient.

^b ^Physical activity characteristics presented in the table are means and 95% confidence intervals (CI), except for moderate/vigorous activity which is geometric mean and 95% CI.

^1 ^Total activity (questionnaire = MET hr/day (excluding sleep); accelerometer = total counts per day). ρ and kappa compares total METs from the questionnaire with total counts from the Actigraph 7164 accelerometer.

^2 ^Light activity (questionnaire = 1.5-3 METS; accelerometer = 100-1951 counts per minute)

^3^Moderate/vigorous physical activity (questionnaire = > 3 METs; accelerometer = > 1951 counts per minute)

P-value for spearman rank and kappa coefficient is a test of independence between the two data measures. *P < 0.05; **P < 0.001

For the sample as a whole, criterion validity was fair for total activity (ρ = 0.41; P < 0.001: kappa 0.34; P < 0.001) and MVPA (ρ = 0.48; P < 0.001: kappa 0.32; P < 0.001) but weaker for light activity (ρ = 0.17; P < 0.05: kappa 0.08; P = 0.09). In sub-group analyses, this pattern was broadly similar amongst urban and rural participants and men, with greatest reliability seen for MVPA (urban and rural men), and weakest or non-significant reliability seen for light intensity activity (rural, urban and men). Reliability for light intensity activity amongst rural participants was higher than for the population as a whole (ρ = 0.31; P < 0.05; kappa 0.13; P = 0.08) and reliability amongst men was highest for total Activity (ρ = 0.40; P < 0.001; kappa 0.33; P < 0.001). For women, only self-reported light intensity activity was significantly correlated using the criterion method, (ρ = 0.45; P < 0.05; kappa 0.24; P < 0.05).

### Construct Validity

**Figure 2:**
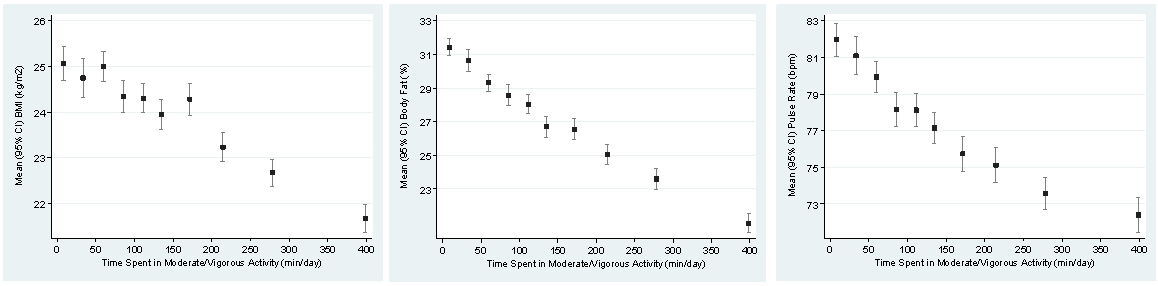
**Means and 95% confidence intervals of BMI, Percent Body Fat and Pulse Rate for deciles of moderate/vigorous activity**.

Total Activity (total METs) was associated with percent body fat and pulse rate, independent of sex, age, migrant status and after mutually adjusting for participation in sedentary, light and MVPA. Individuals in the highest tertile of total activity (total METs) had decreased body fat of 2.09% (95% CI -2.54 to -1.64) and decreased pulse rate of 3.21 bpm (95% CI -4.08 to -2.34) compared to individuals in the lowest tertile of total Activity. Time spent in sedentary activity was also associated with increased BMI and percent body fat, after adjusting for potential confounders. Individuals in the highest tertile of self-reported sedentary time had an increased BMI of 0.64 kg/m^2 ^(95% CI 0.29 to 0.99; P < 0.001) and increased percent body fat 1.38% (95% CI 0.83 to 1.90; P < 0.001) compared to individuals in the lowest tertile of sedentary time. No significant association was observed between time spent sedentary and pulse rate. A reverse pattern was seen for MVPA where individuals in the highest tertile had a decrease in BMI of 0.84 kg/m^2 ^(95% CI -1.16 to -0.52; P < 0.05), decreased body fat of 1.71% (95% CI -2.19 to -1.24; P < 0.001) and a decrease in pulse rate of 3.27 bpm (95% CI -4.20 to -2.34; P < 0.001) compared to the reference group. Figure 2 presents means and 95% confidence intervals of BMI, percentage body fat and pulse rate against deciles of moderate/vigorous activity. No significant associations were found between light activity and percent body fat; BMI or pulse rate.

Rural/urban analysis suggested that stronger associations were seen in rural participants for BMI and percent body fat by sedentary and MVPA intensity. Rural participants who reported the highest amount of total activity (MET hr/day) had a decreased BMI of 1.37 kg/m^2 ^(95% CI -1.87 to -0.87; P < 0.001), decreased body fat of 2.81% (95% CI -3.60 to -2.02; P < 0.001) and decreased mean pulse rate of 3.60 bpm (95% CI -5.11 to -2.08) compared to those in the least active group. Similar findings to those for the sample as a whole were seen for sedentary activity and MVPA although not for light activity in rural participants. Construct validity in urban participants was seen for total activity and MVPA in association with body fat and pulse rate although not for sedentary or light activity or for BMI.

Construct validity varied between men and women (data not shown). Stronger associations were seen for men between BMI, body fat, pulse rate and total and sedentary PA compared to women. In contrast, time spent in MVPA was significantly associated with BMI and pulse rate in women, but not in men. Women who reported the highest amount of time in MVPA (tertile 3) had a decreased BMI of 0.66 kg/m^2 ^(95% CI -1.27 to -0.66; P = 0.03) and decreased pulse rate of 3.71 bpm (95% CI -5.25 to -2.16; P < 0.001).

## Discussion

The results of this study for adults in India show evidence of reliability for the IMS-PAQ, with good intraclass correlation and kappa statistics between baseline and retest. The validity coefficients and associations produced between total activity/activity intensity and theoretical constructs of PA were in agreement with those predicted, providing evidence of construct validity for the IMS-PAQ. These findings suggest that the IMS-PAQ is valid for ranking individuals based on reported PA within this population but that further research may be needed for urban residents and women. This study has constructed categories of PA based upon reported time in different activity intensities and used them to predict associations with relevant health outcomes (BMI, percent body fat and pulse rate) in order to provide a more thorough assessment of the validity of the questionnaire.

The results show that for the sample as a whole the IMS-PAQ has good reliability with intra-class correlations ranging from 0.42 to 0.88, (kappa 0.37 to 0.68) for total activity, television viewing and all activity intensities for those tested within one month. These findings are comparable with previously published values from reliability studies in other LMICs (where the International and Global Physical Activity Questionnaires (IPAQ and GPAQ) recalled activity data over last-week), [7,9] and for Pima Indians in north America, [29] although lower than values obtained from a Sub-Saharan PAQ based in Cameroon, [30] perhaps because in this latter study, test-retest period was restricted to 10-15 days rather than ≤1 and > 1 month. When stratified by urban/rural residency and gender, variations in correlation were seen. No discernable difference in reliability was seen between urban/rural participants, a finding that was discordant with other studies where rural residents display weaker reliability [7,9,30] and in part may be explained by the comparatively higher proportion of males (81%) in the rural sub-sample who tend to provide more reliable data than women, which has been reported previously in both HIC and LMIC [9,30,31].

Results for criterion validity showed that the relationship was good for total activity and MVPA for the sample as a whole (ρ = 0.41-0.48), although not for light activity (ρ = 0.18). These findings held true across sub-groups and were broadly concordant with previously published data from studies in South Africa and China where agreement varied by activity intensity [9]. The stronger correlations seen for men were expected as reported elsewhere [31]. Analysis by urban/rural residency showed stronger correlation for rural participants, a finding that has been noted in other LMICs [9].

Within this study the IMS-PAQ consistently overestimated time spent in MVPA compared to the Actigraph 7164^® ^uniaxial accelerometer, an issue which has been reported elsewhere [32]. This overestimation may in part be due to the context specific nature of activities undertaken within both urban, but particularly rural India which often require considerable upper-body motion (e.g. labour-intensive farming practices). Uniaxial accelerometers are restricted by design in their ability to pick up all body movements and so activities, which involve a high degree of upper-body motion and horizontal movement, may not have been effectively captured [14]. The IMS-PAQ overestimation may represent a bias of the accelerometer rather than of the questionnaire.

Reliability and validity was lower for women than men within this study. In part this may be explained by the lower levels of female education which have previously been associated with poorer criterion validity [9] and socio-cultural conditions whereby a woman's occupation has historically been household chores (88% women and 1% of men in this sub-study reported housework as their occupation). These activities are typically more varied in nature (and often occur concurrently e.g. child care and cooking) than manual or professional occupations, (which generally focus on one main activity), providing greater difficulty in accurate recall and potential for misreporting. Furthermore, evidence from India has previously established that perceptions of activity intensity differ by gender [10]. Additionally the low status of women, particularly in rural areas, may result in their considering work outside home (on their own farms) as unimportant and thus underreporting it. These issues could have added to the poor associations seen among women in particular.

### Strengths and limitations

This paper examines the IMS-PAQ as an instrument designed to assess PA in both rural and urban Indian populations. The broad and diverse nature of the IMS which contains clinical, anthropometric and lifestyle data across four regions of India is one of its main strengths providing theoretical constructs associated with PA on 7,000 participants from which construct validity could be effectively established within sub-groups (men/women, urban/rural). The PAQ, specifically designed for India, allowed participants to report on up to 42 separate activities. Whilst providing rich and diverse data on habitual activity over the last one-month, the open-ended nature of the IMS-PAQ required laborious and time-consuming data cleaning. The open-ended nature of the IMS-PAQ enabled participants to report on any activity undertaken which in turn permitted the over-reporting of daily time spent active. Subsequent adjustments for over and under-reporting of time spent active within the IMS-PAQ allowed comparisons to be drawn between individual participants and migrant groups. Repeating the IMS-PAQ on 5% of the main study and applying motion sensors to over 150 participants enabled reliability and criterion validity to be established. The smaller sample size and greater proportion of urban participants and men within the criterion subsample means results presented may not be generalisable to the study population as a whole. Stratifying reliability to ≤1 and > 1 month and criterion validity to ≤10 hours of activity for at least 4 days, resulted in small sample sizes for the sub-studies making it difficult to separate out gender differences accordingly. Additionally, the allocation of MET values for self-reported activities was based on previously published data primarily from HIC settings, rather than measured scores for each individual. The clustered nature of the sample by sib-pairs, whilst accounted for within construct validity, may have underestimated CI presented for the reliability and construct validity sub-studies.

## Conclusion

Questionnaires such as the IPAQ and GPAQ designed to capture PA at a population level are often too prescriptive in style to fully encompass socio-cultural differences, thus preventing detailed information being gathered on country-specific activities across multiple-domains. Questions within the IPAQ and GPAQ are more limited in scope and ask participants to report back on the perceived intensity of activities undertaken rather than reporting specific activities. Designing an instrument to accurately and reliably measure PA within LMICs, in particular the change in activity levels and patterns as individuals migrate from rural to urban areas, could provide an important contribution to PA monitoring and assessment.

In our current investigation, we demonstrated that the IMS-PAQ has good test-retest reliability and is able to rank individuals based upon their level of PA. The values obtained are comparable to those from the IPAQ and GPAQ despite the contrasting scope of the questionnaires. We have also shown that the IMS-PAQ is reliable and valid for comparing urban and rural populations, especially for total activity (MET hr/day) and MVPA. These findings held true for men although reliability and validity was lower in women. We therefore recommend that future research efforts continue to examine gender differences in reporting activity, as well as continue to enhance the reliability of the IMS-PAQ given the continual and expanding prevalence of NCDs in India.

## Competing interests

All authors declare that they have no relationships with companies that might have an interest in the submitted work in the previous 3 years; their spouses, partners, or children have no financial relationships that may be relevant to the submitted work; and have no non-financial interests that may be relevant to the submitted work.

## Authors' contributions

SK, BAV, MV, AK, KSR, DP and SE contributed to the design and conduct of the study. UE, TC, HK and RS designed the analysis of the study. RS conducted the analysis. RS drafted the manuscript. HK, UE and SE helped write the manuscript. All authors provided feedback on the manuscript and approved the submitted version.
